# Planned iliohypogastric neurectomy for prevention of chronic pain after inguinal hernia repair

**DOI:** 10.1007/s10029-025-03283-1

**Published:** 2025-03-13

**Authors:** Kazım Gemici, Ersin Özeren

**Affiliations:** 1 Faculty of Medicine, Head of Gene Ral Surgery Department, Aksaray University, Aksaray, Turkey; 2 Faculty of Medicine, Neurosurgery Department, Aksaray University, Ankara University Graduate School of Health Sciences Department of Clinical Anatomy, Ankara, Turkey

**Keywords:** Inguinal hernia, Herniorrhaphy, Groin, Pain

## Abstract

**Purpose:**

The present study aimed to investigate the effect of planned ilohypogastric neurectomy (IHPN) in preventing chronic postoperative inguinal pain (CPIP) after anterior herniorrhaphy (AH).

**Materials and methods:**

This prospective, randomized study was conducted between 2016 and 2023. Emergency and incarcerated hernia cases, bilateral inguinal hernias, cases with complications such as postoperative hematoma infection, cases in which the neuroanatomy of the inguinal region was unintentionally damaged, femoral hernias, and paraplegic patients with loss of sensation who underwent anterior inguinal herniorrhaphy with prolene mesh were excluded, and the remaining 1375 patients were included in this study. The patients were randomized into preoperative control and study groups. After further excluding 247 patients (18%) in whom the IHN could not be identified during the operation, 82% of the 1375 patients (n = 1128) were included in this study. In the control group, the RCA segment of the IHN that would remain under the mesh was preserved (control group = G1 = 534). The second group in which this segment of the RCA was excised comprised the study group (G2 = 594). Two groups were prospectively followed and pain scores (PS) were recorded regularly with a 4-point scale. The average follow-up period was 15 months (range 11–19 months) in G1 and 14 months (range 10–18 months) in G2.

**Results:**

The number of patients with CPIP was 68 in G1 and 19 in G2, this difference was found to be statistically significant (p: < 0.001). There were 19 patients in G1 and 41 patients in G2 with loss of sensation in the operation area, and a significant difference was detected in this respect (p = 0.012).

**Conclusion:**

The rate of developing CPIP was significantly lower in patients who underwent IHPN during anterior herniorrhaphy than in those who did not undergo IHPN. The subjective nature of the pain sensation complicates measuring and scoring this sensation and methodologically limits the study.

## Introduction

Groin pain after inguinal herniorrhaphy is a common symptom, although it is expected to decrease within 2 months. Some patients may continue to experience pain at different levels, which may negatively impact their daily activities and sexual life. Open inguinal herniorrhaphy is one of the most commonly performed operations, with one million operations performed annually in the United States and 20 million across the world. To diagnose chronic pain, the pain must last longer than 3 months without any other cause [1]. Several factors play a role in the etiology of pain, including whether the operations are performed open-access or laparoscopically, whether the nerves are protected, the type of mesh, the fixation suture, the presence of pain before the operation, the development of complications, gender, and the development of neuroma. Moreover, there are continuing discussions on the incidence, terminology, pathogenesis, and treatment strategy in patients diagnosed with CPIP in the literature [2–4]. Although pain after herniorrhaphy is generally expected to decrease within 2 months, the abovementioned factors affect the development of CPIP, with the most important factor being the involvement of the ilioinguinal (IIN), iliohypogastric (IHN), and genitofemoral (GFN) nerves that constitute the neuroanatomy of the lower abdomen and groin region. These nerves originate from the lumbar plexus and provide cutaneous sensory innervation to the groin and upper thigh [5, 6]. These nerves are inevitably encountered during anterior open hernia surgery. It has been reported that half of the patients continue to experience varying degrees of pain after 1 year of follow-up, whereas 15% continue to experience moderate or severe pain that limits their daily activities [7]. Although the incidence of CPIP varies significantly in several studies and according to guidelines, the reported rate is 0.7%–75%; however, the prevalence of CPIP is severe enough to limit daily activities, and work power is reported as 0.5%–6% [4, 8, 9]. This difference in prevalence reflects the complexity of the definition and pathogenesis of CPIP. The effects of planned neurectomies on this pain have been extensively investigated [10–13].

### Anatomy of the IHN

The IHN is a mixed branch of the lumbar plexus. It originates from the anterior/ventral ramus of the L1 spinal nerve root as a single trunk with the IIN. The IHN emerges from the upper lateral border of the psoas major muscle, behind the kidneys, and in front of the quadratus lumborum muscle, and begins its course on the posterior abdominal wall, then courses between the anterior abdominal muscles, and enters the anterior abdominal wall obliquely. When the IHN reaches the anterior abdominal wall, it enters the muscle transversus abdominis (MTA) posteriorly at a level just above the iliac crest and gives off two branches as it courses between the MTA and the muscle obliquus internus abdominis (MOI), viz., the anterior cutaneous and the lateral cutaneous branches.

### Anterior cutaneous branch (ramus cutaneous anterior, RCA)

The anterior cutaneous branch receives sensory branches from the skin of the hypogastric region. The somatomotor branches continue to pass between the MTA and MOI and innervate both muscles. Approximately 2 cm medial to the anterior superior iliac crest, it passes above the MOI and courses in the area where the mesh will be laid, and approximately 3 cm above the superficial inguinal ring, it passes through the aponeurosis of the musculus externus abdominis (MOE) and reaches the subcutaneous tissue.

### Lateral cutaneous branch (ramus cutaneous lateralis, RCL)

The lateral cutaneous branch penetrates the MOI and MOE at a level just above the iliac crest and passes subcutaneously. It then distributes to the skin in the middle and outer parts of the gluteal skin. The IHN, a mixed nerve, provides both motor and sensory innervation to the abdominal muscles and sensory innervation to the skin of the posterolateral gluteal and suprapubic regions.

In anterior herniorrhaphy, before the RCA pierces the MOE, approximately 3–5 cm of the lateral part of the conjoint tendon (CT) remains under the mesh to be laid, and 5% of the IHN progresses inside the MOI; therefore, because it is not visible, it can be compressed when applying a suture or stapler, which plays a vital role in the development of CPIP. It has been reported that in open inguinal hernia surgery, the IHN remains under the mesh and is exposed to inflammatory reaction and compression, causing myelin damage and edema in the nerve [14].

## Materials and methods

**Fig. 1:**
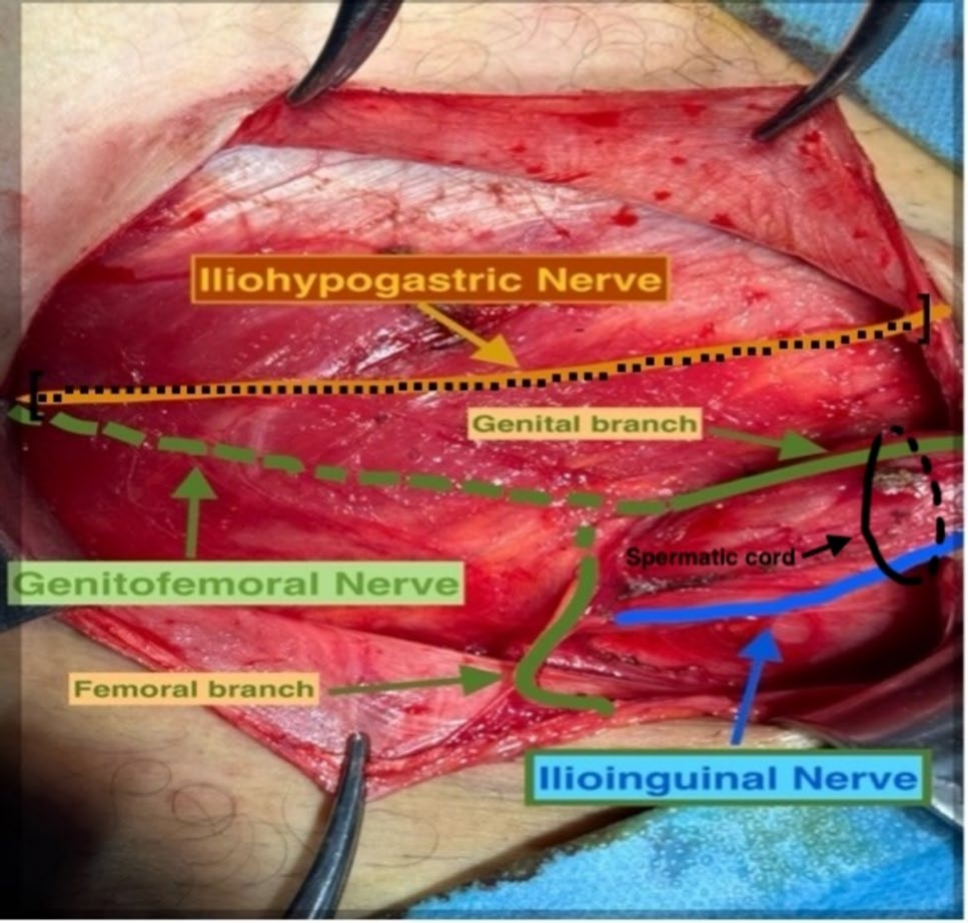
Inguinal neuroanatomy, [….] anterior branch of the excised IHN

This prospective, randomized study was conducted between 2016 and 2023. Emergency and incarcerated hernia cases, bilateral inguinal hernias, cases with complications such as postoperative hematoma infection, cases in which the neuroanatomy of the inguinal region was unintentionally damaged, femoral hernias, and paraplegic patients with loss of sensation who underwent anterior inguinal herniorrhaphy with prolene mesh were excluded, and the remaining 1375 patients were included in this study. The patients were randomized into preoperative control and study groups. After further excluding 247 patients (18%) in whom the IHN could not be identified during the operation, 82% of the 1375 patients (n = 1128) were included in this study. In the control group, the RCA segment of the IHN that would remain under the mesh was preserved (control group = G1 = 534). The second group in which this segment of the RCA was excised comprised the study group (G2 = 594). In both groups, the IIN and GFN were preserved when seen, whereas the RCA branch of the IHN was preserved in G1. In G2, the RCA segment between the point where it exits the MOI and the point where it pierces the MOE fascia was excised, and its proximal and distal ends were ligated with absorbable sutures to prevent it from remaining under the mesh or being compressed by the suture (Fig. 1).

In the present study, the operations were performed by the same surgeon in both groups using the same technique and materials except for IHPN. As a standard, we fixed the mesh with CT and 2/0 round absorbable single sutures (5–8 passes with 2/0 polyglactin) to the inguinal ligament (IL) at intervals. Informed consent forms were obtained from the patients in both groups. All procedures performed in studies involving human participants were conducted according to the ethical standards of the institutional and/or national research committee and the 1964 Declaration of Helsinki and its later amendments or comparable ethical standards. This study was approved by the ethics committee of Mevlana University, Faculty of Medicine (2015/09–134). Demographic data of the patients were obtained, and preoperative pain was evaluated and recorded using a 4-point scale (0 = none, 1 = mild, 2 = moderate, and 3 = severe) at the end of 1, 2, and 3 months. In most of the literature, postoperative groin pain is defined as no pain, moderate pain that does not affect daily activities, and severe pain that prevents daily activities. Therefore, since the four-point pain scoring system is thought to be more appropriate for this definition in the literature, this scoring system was used. Mild pain refers to insignificant pain in the operation area that does not increase with activities such as walking and climbing stairs, moderate pain indicates pain that becomes apparent with walking but does not restrict daily activities, and severe pain implies pain that completely prevents daily activities. All mild-to-severe pain scores expressed by the patients at the end of the 3rd month were defined as CPIP. The patients came for monthly check-ups and were examined. Patients whose pain did not subside at the end of the 3rd month were diagnosed with CPIP. Patients were planned to be followed up for at least 1 year. The average follow-up period was 15 months (range 11–19 months) in G1 and 14 months (range 10–18 months) in G2. Pain scores of the patients were reevaluated at 6 and 12 months, and the analgesics used were recorded along with patients who had a loss of sensation at the end of the 1st year. Clinical evaluation of the sensory branch of the hypogastric nerve was performed by postoperative sensory examination. The sensory region of the hypogastric nerve corresponds to the T12-L1 dermatomal area. Therefore, a sensory examination was conducted on the skin tissue just above the IL on the surgical side. This examination was bilateral, and the patient was asked about the difference between the two sensations and the side that felt less sensation, and the result was recorded.

Nerve block (NB) was performed by the same neurosurgeon after the 12th month in patients with CPIP who did not respond to medical treatment. Triple (in G1) or double (in G1/G2) neurectomy was performed in patients who did not respond to NB. Pain score was selected as the primary outcome for calculating the sample size. To detect a difference of 0.25 units in pain scores between the control and experimental groups with statistical significance, the required sample size was calculated based on a two-tailed t-test. Assuming a significance level of 0.05 and a minimum power of 0.80 (maximum Type II error of 0.20), the required number of participants per group was determined as 252. The Mann–Whitney U test was used to compare the variables obtained from measurements between independent groups, and the chi-square and Fisher’s exact tests were used to evaluate the relationship or differences between groups in terms of categorical variables. Furthermore, risk criteria for group factors were determined using the univariate logistic regression method, and results were expressed using regression coefficient, standard error, odds ratios, and confidence intervals. Percentages and frequency distributions were provided for categorical variables as descriptive statistics, and mean ± SD and median (minimum–maximum) values were provided for continuous variables. Statistical analyses were conducted using the IBM SPSS Statistics for Windows, Version 26.0 program, with p < 0.05 accepted as the statistical significance limit.

## Results

Table 1 shows the demographic data of the patients, distribution of hernia types, and presence of preoperative pain. There was no significant difference in terms of pain between G1 and G2 at the 1st and 2nd months of PO (p < 0.127 and p < 0.541, respectively). In the 3rd month of PO, there were 68 and 19 patients diagnosed with CPIP in G1 and G2, respectively, and this difference was significant (p < 0.001). Moreover, this difference was significant in favor of G2 between G1 and G2 at the 6th and 12th months (p < 0.001) (Table 2). In the 12th month of PO, there were 16 and 2 patients with pain in G1 and G2, respectively.

**Table 1:** Preoperative patient information

		G1 (Control)		G2 (IHPN)		*P*
		N	%	n	%	
Gender	Male	492	92,1	549	92,4	0,856
	Female	42	7,9	45	7,6	
Hernia type	I	393	73,6	430	72,4	< **0,001**
	D	83	15,5	141	23,7	
	M	58	10,9	23	3,9	
Preoperative pain	None	180	33,7	216	36,4	0,351
	Available	354	66,3	378	63,6	

*I* indirect, *D* direct, *M* mix, *p* p value, *IHPN* Iliohypogastrik planned neurectomy

**Table 2:** Postoperative pain

		G1 (Control)		G2 (IHPN)		*p*
		n	%	n	%	
OD need for T		53	9,9	40	6,7	0,052
PO 1. Month P	None	416	78	471	79,3	0,127
	Available	118	22	123	20,7	
PO 2. Month P	None	457	85,5	511	86	0,541
	Available	77	14,5	83	14	
PO 3. Month P	None	466	87,3	575	96,8	< **0,001**
	Available	**68**	12,7	**19**	3,2	
PO 6. Month P	None	491	92	587	98,8	< **0,001**
	Available	43	8	7	1,2	
PO 12. Month P	None	505	94,5	587	98,8	< **0,001**
	Available	29	5,5	7	1,2	

Bold values indicate statistically significant values

*PO* postoperative, *OD* operation day, *P* pain, *PS* pain score, *T* tramadol hydrochloride, *p* p value, *IHPN* Iliohypogastrik planned neurectomy

According to the univariate logistic regression analysis, the probability of experiencing chronic pain in the 3rd month of PO in G1 patients (odds) was approximately 4.41 times higher than that in G2 patients (2.617–7.451), and this increase in risk was statistically significant (p < 0.001) (Table 3). According to the 4-point scale, patients with pain scores of 1, 2, and 3 were diagnosed with CPIP. In G1, there were 23 patients with mild pain (score 1), 27 patients with moderate pain (score 2), and 18 patients with severe pain (score 3), and in G2, the respective numbers of patients were 11, 7, and 1.

**Table 3:** Probability of pain in the control group

	B ± S.E	Odds ratio (%95 Confidence interval)	p
G1	1,485** ± **0,267	4,416 (2,617 – 7,451)	** < 0,001**

Bold values indicate statistically significant values

*B* logistic regression coefficient, *SE* standard error

**Graph 1:**
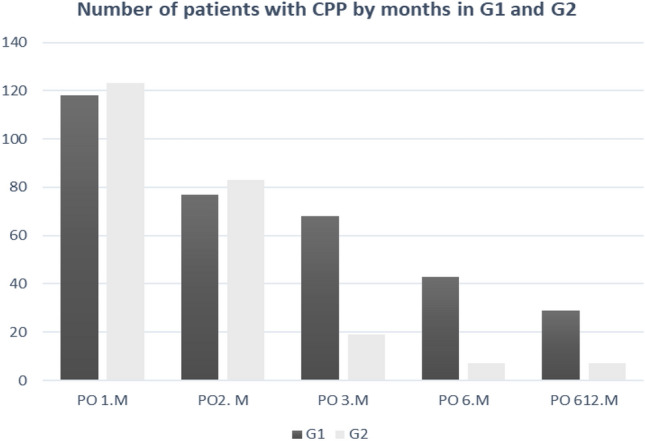
Number of patients with CGP by month, based on groups (*PO *Postoperative*, M *Month)

No significant difference was observed between patients with preoperative groin pain and those with CPIP in both groups (p < 0.351). On the day of operation, we routinely applied any one of NSAIDs, paracetamol (PST) and methimazole sodium or any two in combination for analgesia to our patients, and for patients whose pain does not subside with these analgesics, tramadol (T) was administered twice a day. There was no significant difference between the number of patients requiring OD tramadol in both groups (p < 0.052). The number of patients with CPIP according to month on a group basis is presented in Graph 1, which indicates that pain was significantly less in the IHPN group after the 3rd month. NSAIDs, PST, and T were administered to patients diagnosed with CPIP in the first stage, and then pregabalin (PG) was administered alone or in combination with the analgesics in the first stage to patients in whom these analgesics were insufficient (Table 4).

**Table 4:** PO medical and surgical treatment

		G1(Control)		G2 (IHPN)		p
		n	N %	n	N %	
PO 3. Month Medical treatment	PST	2	2,9	3	15,8	** < 0,001**
	NSAI	24	35.3	8	42,0	
	NSAI + T	4	5,9	4	21,0	
	PG	5	7,4	3	15,8	
	PG + NSAI	4	5,9	0	0,0	
	PG + T	29	43,0	1	5,3	
PO 12. Month Medical treatment	NSAI	14	48,3	3	42,9	1,000
	PG	12	41,3	2	28,9	
	PG + T	3	10,4	2	28,9	
PO 12. Month Nerve Block	NB	9	31	2	28,9	0,495
Surgical treatment	TN-BN	3	33,3	1	50,0	1,000
Sensation change or loss	None	515	96,4	553	93,1%	**0,012**
	Available	19	3,6	41	6,9%	

Bold values indicate statistically significant values

*PO* postoperative, *PST* paracetamole, *PS* pain score, *T* tramadolhydrochloride, *NSAI* nonsteroidal anti-ınflammatory, *PG* pregabalin, *NB* nerveblock *TN* triple neurectomy, *p* p value

In G1, 29 patients whose pain continued at 12 months were diagnosed with groin pain due to lumbar disc herniation. Surgery was performed by the same neurosurgeon, and the groin pain resolved. In G2, there were 41 (6.9%) patients with loss of sensation, and in the control group, this number was 19 (3.6%). Loss of sensation was significantly higher in the IHPN group (p = 0.012). The median PS scores among the groups at 3 months postsurgery are presented in Table 5.

**Table 5:** Median PS at 3 months between groups

	G1(Control)		G2 (IHPN)		p
In the 3rd month PS	1.92 ± 0.78	2.00 (1.00–3.00)	1.47 ± 0.61	1.00 (1.00–3.00)	**0.024**

Mean ± S.Deviation and Median (Min.–Max.)

*PS* pain score

We also determined the distribution of the number of patients whose pain was zero (PS = 0) and whose pain was not zero (PS > 0) in the 12th month of PO in controls according to the groups and found that the difference between the distributions was statistically significant (p = 0.003). Accordingly, the number of patients whose pain was zero in G2 was significantly higher than that in the control group (Table 6).

**Table 6:** Number of patients whose pain disappeared at 12 months of PO

	G1(control)		G2(IHPN)		P
	n	N%	n	N%	
PS > 0	50	73,5	7	36.8	**0,003**
PS = 0	18	26,5	12	63,2	
Total	68	100,0	19	100,0	

*PO* Postoperative *PS* Pain score, *IHPN* Iliohypogastric planned neurectomy

## Discussion

Inguinal hernia operations are one of the most frequently performed procedures in the world (60%), and approximately 0.7%–75% of patients develop CPIP at different degrees. In 0.5–6% of these patients, this pain can be severe enough to negatively impact daily activities and sexual functions [4, 15, 16]. Inguinal pain after open herniorrhaphy is generally expected to subside within 2 months. However, a survey of 2500 patients in Sweden reported that CPIP continued at different degrees in 30% of patients even in the 2nd and 3rd years after herniorrhaphy, and in 11%–14% of them, the pain was moderate and severe and prevented daily activities [17]. Acute pain is nociceptive due to the inflammatory response in the surgical site and can last for 6–8 weeks. Chronic pain is neuropathic and develops due to abnormal neural activity for reasons such as complete or partial nerve injuries without inflammation, neuroma formation, use of nonabsorbable sutures, and nerve compression by stapler [18]. In the study group, the RCA segment of the IHN under the mesh was excised to eliminate mesh-nerve interaction. In one of the studies supporting this hypothesis in the literature, an investigation of the pathogenesis of CPIP reported that the contact of the nerves in the operation area with the mesh resulted in myelin sheath damage, edema, and fibrosis development around the nerve as an important factor [14, 19].

Although it is difficult to distinguish between nociceptive and neuropathic pain, the International Association for the Study of Pain (IASP) defines nociceptive pain as pain that is not neural, that results from actual or threatened tissue damage, and that results from the activation of nociceptors. Conversely, neuropathic pain is defined as pain that results from a lesion or disease of the somatosensory nervous system [&Merskey H, Boogduk N. Classification of Chronic Pain: Descriptions of Chronic Pain Syndromes and Definition of Pain Terms. 2nd ed. Seattle, WA: IASP Press; 1994]. Patients may experience pain-free periods after herniorrhaphy, and CPIP may occur weeks, months, or even years later. The clinical features of the pain are episodic, burning, stabbing, and pricking and can be evoked by a trigger point and may radiate to the scrotum, labia, buttocks, and pelvis. Pain can be triggered by activity, hip hyperextension/high flexion, touching the operation area, coughing, breathing, and even bowel movements and can be relieved by lying down [20–23]. The lower abdomen and groin nerves, i.e., IIN, IHN, and GFN, can be damaged voluntarily or involuntarily during the operation, which plays a vital role in the development of CPIP. Several studies have demonstrated the relationship between CPIP development and IIN and IHN. For instance, a study compared IIN and IHN blocks with the block applied to the MTA plane and found that the IIN and IHN blocks were superior in preventing CPIP [24]. In most of those studies, the visual–verbal numerical rating scale system was used for scoring pain in patients. In most of the literature, postoperative groin pain is defined as no pain, moderate pain that does not affect daily activities, and severe pain that prevents daily activities [25]. Therefore, since the four-point pain scoring system is thought to be more appropriate for this definition in the literature, this scoring system was used in our study. Some risk factors for CPIP were determined in retrospective studies, such as young age, preoperative pain, early severe postoperative pain, female gender, postoperative complications, recurrent hernia, anterior approach, and IHN excision [17, 26]. However, in our study, the rate of CPIP development was lower in patients who underwent IHPN. It has been emphasized that pathological stimuli resulting from damage to the inguinal nerves, inflammation, and contact with the mesh during herniorrhaphy increase the development of CPIP; therefore, prophylactic neurectomy reduces this pain development [23]. Similar to that in our study, three studies including 270 patients who underwent IHPN reported that fewer patients in the neurectomy group developed CPIP than those in the non-neurectomy group (risk 0.69, 95%, CI 0.52–0.90), and none of the patients who underwent neurectomy developed significant loss of sensation [27]. Furthermore, another 11 randomized controlled studies including 1033 patients who underwent prophylactic neurectomy of the IIN reported a significant decrease in the number of patients who developed CPIP [10].

During Lichtenstein herniorrhaphy, especially because the anterior branch of the IHN courses on the posterior wall of the inguinal canal, in most cases, it remains under the mesh during fixation on CT and is exposed to a serious inflammatory reaction or can be compressed by the fixation suture. The identification rate of the three nerves has been reported to be 70–90%, and complete resection of the damaged nerve was recommended [4]. In our study, the identification rate of IHN was 82%. Results of 23 meta-analyses investigating the effect of mesh on the development of chronic pain showed a prevalence of CPIP development of 2.9% (0–28%) in herniorrhaphy without mesh, whereas this rate was 3.5% (0–16%) in cases where mesh was used. Another meta-analysis including 21 studies emphasized that the weight of the mesh exerted no effect on the development of CPIP [28, 29]. Ultrasound, computed tomography, and magnetic resonance imaging are helpful in eliminating complications such as infection, neuroma, fluid collection, and mesh collection in cases of CPIP that lasts more than 2 months. After excluding nonneuropathic causes, the pain is expected to decrease in 6–8 weeks. The IASP has reported that pain that lasts more than 3 months should be defined as CPIP [30, 31]. Burning, stabbing pain in the area of the influence of the groin nerves, and pain relief with an anesthetic applied to the nerves in this area allow us to diagnose CPIP; however, the lack of pain relief does not exclude the diagnosis of CPIP. NSAIDs and opioids are recommended in the first stage of treatment [27].

NB can be performed on patients whose pain does not subside with medical treatment. It is performed by pain specialists and surgeons using the same method. A mixture of long-acting local anesthetic containing 5–10 ml of 0.25–0.50% bupivacaine and 25 mg hydrocortisone or 20 mg methylprednisolone is injected into the tissue around the affected nerve. If the pain decreases, the treatment is repeated with weekly applications until the relief becomes permanent. If this method fails, percutaneous nerve ablation with phenol or alcohol can be performed; alternatives to this method include radiofrequency and cryoablation. Antiepileptics such as gabapentin and PG and antidepressants such as duloxetine can be preferred before proceeding to surgery. Although there is no full consensus on the timing of surgery, the common opinion among experts is an elapse of at least 1 year. Nerve and mesh excision is the most common surgical procedure, with success rates ranging from 70 to 100%. Guidelines recommend triple neurectomy instead of selective neurectomy, and it is emphasized that 90% of these patients experience relief in CPIP. If the removed mesh is anterior, a new mesh is placed laparoscopically, and if it is posterior, a new mesh is placed anteriorly. Patients who fail surgery may benefit from nerve stimulation, which is in the experimental phase [22, 32, 33]. Other alternative treatments, such as acupuncture, heat application, topical analgesics, and physiotherapy, have been reported to be beneficial in the short term. Surgeons who perform prophylactic neurectomy cut the nerves where they enter the muscle and ligate or cauterize the nerve endings [34]. In our study, the nerve endings were ligated with absorbable sutures. With the increase in the number of inguinal hernia operations across the world, there is also an increase in the number of patients diagnosed with CPIP. Nevertheless, there are still ongoing discussions in the literature regarding the incidence, terminology, pathogenesis, and treatment strategy. A working group consisting of nine international experts who began their study in 2007 determined 6 major questions and 23 subgroups of questions, one of which was “Whether prophylactic neurectomy has an effect on the reduction of CPIP?” The aim of that study was to seek an answer to this question. The working group searched the literature for answers for 1 year. A meeting was held in Rome during April 21–22, 2008, to reach a consensus on this issue. If possible, the three nerves should be preserved; however, in the case of the development of nerve damage during the operation, the damaged nerve segment should be removed to prevent somatosensory complaints. No consensus has been reached concerning the procedure to be performed on the nerve endings (such as ligation and cauterization). As a result of the study by the working group, the rates of CPIP development were 12.5% in cases where sutures were used in mesh fixation and 4.5% in cases where fibrin glue was used. When fixing the mesh medially, it is recommended that its sutures be passed through CT, as there is a possibility of compression of the intramuscular segment of the IHN if the sutures are accidentally passed through the muscle [35, 36]. When CPIP develops in patients who underwent herniorrhaphy, the first choice is medical treatment, and if this fails, surgery is recommended after at least 1 year. Triple neurectomy and removal of mesh are recommended as surgical options in expert hands. The relief rate after a triple neurectomy is 80% [37]. In general, the subjective nature of pain sensation, with the pain perception threshold being different for each individual, and variations in the anatomy of the inguinal nerve result in differences in the diagnosis of CPIP and its regional incidence. Therefore, surgeons performing herniorrhaphy must attempt to find a solution for CPIP, which is extremely difficult to manage, using the literature and their individual experiences. It appears inevitable that the possibility of hypoesthesia will increase to prevent chronic groin pain.

## Conclusion

In herniorrhaphy performed with anterior prolene mesh, the number of patients diagnosed with CPIP in the IHPN group was lower than that in G1 with preserved inguinal neuroanatomy, and this difference was statistically significant. We expect to reduce the number of patients developing CPIP, which is difficult to manage, and contribute to the literature through the present study.
